# Practical impacts of genomic data “cleaning” on biological discovery using surrogate variable analysis

**DOI:** 10.1186/s12859-015-0808-5

**Published:** 2015-11-06

**Authors:** Andrew E. Jaffe, Thomas Hyde, Joel Kleinman, Daniel R. Weinbergern, Joshua G. Chenoweth, Ronald D. McKay, Jeffrey T. Leek, Carlo Colantuoni

**Affiliations:** Lieber Institute for Brain Development, 855 N Wolfe St, Ste 300, Baltimore, MD 21205 USA; Department of Biostatistics, Johns Hopkins Bloomberg School of Public Health, 615 N Wolfe St, Baltimore, MD 21205 USA; Department of Neurology, Johns Hopkins School of Medicine, Baltimore, MD 21205 USA; Department of Psychiatry, Johns Hopkins School of Medicine, Baltimor, MD 21205 USA; Department of Neuroscience, Johns Hopkins School of Medicine, Baltimore, Maryland 21205 USA; McKusick-Nathans Institute of Genetic Medicine, Johns Hopkins School of Medicine, Baltimore, Maryland 21205 USA

**Keywords:** Batch correction, Gene expression, Surrogate variable analysis

## Abstract

**Background:**

Genomic data production is at its highest level and continues to increase, making available novel primary data and existing public data to researchers for exploration. Here we explore the consequences of “batch” correction for biological discovery in two publicly available expression datasets. We consider this to include the estimation of and adjustment for wide-spread systematic heterogeneity in genomic measurements that is unrelated to the effects under study, whether it be technical or biological in nature.

**Methods:**

We present three illustrative data analyses using surrogate variable analysis (SVA) and describe how to perform artifact discovery in light of natural heterogeneity within biological groups, secondary biological questions of interest, and non-linear treatment effects in a dataset profiling differentiating pluripotent cells (GSE32923) and another from human brain tissue (GSE30272).

**Results:**

Careful specification of biological effects of interest is very important to factor-based approaches like SVA. We demonstrate greatly sharpened global and gene-specific differential expression across treatment groups in stem cell systems. Similarly, we demonstrate how to preserve major non-linear effects of age across the lifespan in the brain dataset. However, the gains in precisely defining known effects of interest come at the cost of much other information in the “cleaned” data, including sex, common copy number effects and sample or cell line-specific molecular behavior.

**Conclusions:**

Our analyses indicate that data “cleaning” can be an important component of high-throughput genomic data analysis when interrogating explicitly defined effects in the context of data affected by robust technical artifacts. However, caution should be exercised to avoid removing biological signal of interest. It is also important to note that open data exploration is not possible after such supervised “cleaning”, because effects beyond those stipulated by the researcher may have been removed. With the goal of making these statistical algorithms more powerful and transparent to researchers in the biological sciences, we provide exploratory plots and accompanying R code for identifying and guiding “cleaning” process (https://github.com/andrewejaffe/StemCellSVA). The impact of these methods is significant enough that we have made newly processed data available for the brain data set at http://braincloud.jhmi.edu/plots/ and GSE30272.

**Electronic supplementary material:**

The online version of this article (doi:10.1186/s12859-015-0808-5) contains supplementary material, which is available to authorized users.

## Background

High-throughput experiments are commonplace in molecular biology and aim to identify genomic measurements associated with clinical phenotypes and biological mechanisms. Microarrays and next generation sequencing are popular measurement tools for these experiments and assay tens of thousands of genes at once. Typically, data normalization and preprocessing approaches reduce technical variability [1, 2] but there often remain high levels of systematic heterogeneity in the data which can obscure biological phenomena under study. Because its impact is often severe, understanding of such heterogeneity should be an integral part of processing and exploration of genomic data.

Much of this underlying variability is observed to be systematic with the order in which samples are processed, and therefore is commonly referred to as “batch effects” [3]. There are currently two general classes of “batch” correction methods: those that use linear modeling when batches are known or assumed (e.g. ComBat [4]) and those that attempt to identify and control for potential batch effects (e.g. surrogate variable analysis [SVA] [5], remove unwanted variation (RUV) [6], among others). While uncorrected “batch effects” still appear in published high-throughput data, an additional, more subtle, yet perhaps more common issue has arisen: the incorrect or imprecise definition of biological enquiry during “cleaning” of genomic data, resulting either in the removal of important biological signal, or the retention of unwanted latent variability. It is important to note that all of these algorithms depend on well-designed studies to properly identify these “batch effects”, i.e. where the outcome of interest is balanced across potential batches, as in randomization procedures—otherwise, it is difficult to attribute variance in the data to “batch” or biology of interest [7].

The first class of batch correction methods may miss artifacts due to biology, and unannotated technical variation, while the second class of factor-based estimation may remove biological variation of interest. Here we present three illustrative data analyses using SVA (as a surrogate for almost any factor-based approach) and describe how to perform artifact discovery in light of natural heterogeneity within biological groups, secondary biological questions of interest, and non-linear treatment effects. We do this using two publicly available gene expression datasets, one from differentiating pluripotent cells, and another in the developing and aging human brain. Our analysis indicates that artifact discovery is an important component of high-throughput analysis pipelines but caution should be exercised in supervising the discovery of artifacts to avoid removing biological signal of interest. In particular, researchers should be aware that many biological effects of potential importance can be removed if they are not explicitly protected during the cleaning process.

## Methods

### A summary of SVA

Regardless of data processing methods used, an explicit definition of the precise biological question under study is particularly crucial in genomics investigation. Using SVA formalizes this process in that this “biological model” is an explicitly defined mathematical model passed to SVA. Effects specified in this model are preserved while systematic heterogeneity that affects many measurements unrelated to these effects are identified and subsequently adjusted for in subsequent statistical analyses. This approach has previously been shown to result in more accurate and stable gene rankings, improved false discovery estimation and correct p-value distributions [5]. Under this framework, the biological effects interrogated in the data must be limited to those specified in this model as passed to the SVA algorithm. If this is not the case, effects of interest may be treated as latent heterogeneity and removed from the data. The impact of researchers’ conception of the biological enquiry on the nature of possible discovery should not be underestimated in this process.

SVA’s purely data-driven methods for the estimation of and adjustment for unwanted systematic variance take particular advantage of the breadth of high-dimensional genomics data. The algorithm does not require a priori information about what variables measured by the researcher might represent a “batch effect”. By using the structure of thousands of measures uncorrelated to the effect under study, SVA estimates unwanted effects and allows sculpting of a dataset to focus on an explicitly defined biological effect. This is important as commonly-used “batch” variables, such as microarray scan/hybridization date, are likely surrogates for unmeasured variables that are better estimated by the data themselves [3]. To determine the number of surrogate variables (SVs) to estimate (where more SVs reflects a higher degree of correction applied to the data) the SVA algorithm can take user input, or use an automated approach via permutation testing to estimate the number of SVs present in the data. The correct usage of SVA has the potential to increase statistical power when analyzing experimental data, but note that while increasing the number of SVs reduces the variability in the dataset, it may also reduce variability in the direction of the effect of interest based on the iterative algorithm used to estimate the SVs.

To best assess the biological effect of interest, all estimated SVs, along with the defined “biological model” are included for the adjustment of data in downstream differential expression statistical analyses [8]. We note that the SVA algorithm can permit correlation between these SVs and the outcome of interest [5]. Model selection that removes surrogate or measured variables will lead to *p*-values that are smaller than their true value (anti-conservative biases) [5], greatly increasing probability of identifying false positives. Additionally, SVs can be regressed out of the data to obtain “cleaned” data for visualization (as we do in this report), however differential expression statistics should not be performed on this “clean” data, as this too can lead to anti-conservative bias resulting from between-sample correlation being introduced by regressing out the SVs and from inflating variance partitioning related to the effect of interest, as the total variance of the system has been reduced without being taken into account during the linear modeling. We also suggest incorporating a priori biological data, when available, into the evaluation of data “cleaning” by any method, with the goal of using known biology as an additional guide in this process (as we also do in examples here).

## Results

### Batch correction increases ability to detect defined effects

First, we analyze gene expression data from differentiating pluripotent cells [9] (GSE32923) using the principles outlined above as a guide in applying SVA to focus on particular biological effects in the data. These analyses illustrate how the details of defining biological enquiry in the “cleaning” process impact discovery on both a global and gene-specific level. Human pluripotent cell lines were differentiated towards neuroectodermal and mesendodermal fates. Biological replicates (duplicates) for each cell line in each condition were collected after 8 days of differentiation and hybridized to Agilent G4112F arrays to measure gene expression. Data were preprocessed and normalized using the ‘limma’ R package [10] with background correction and quantile normalization (Additional file 1).

**Fig. 1:**
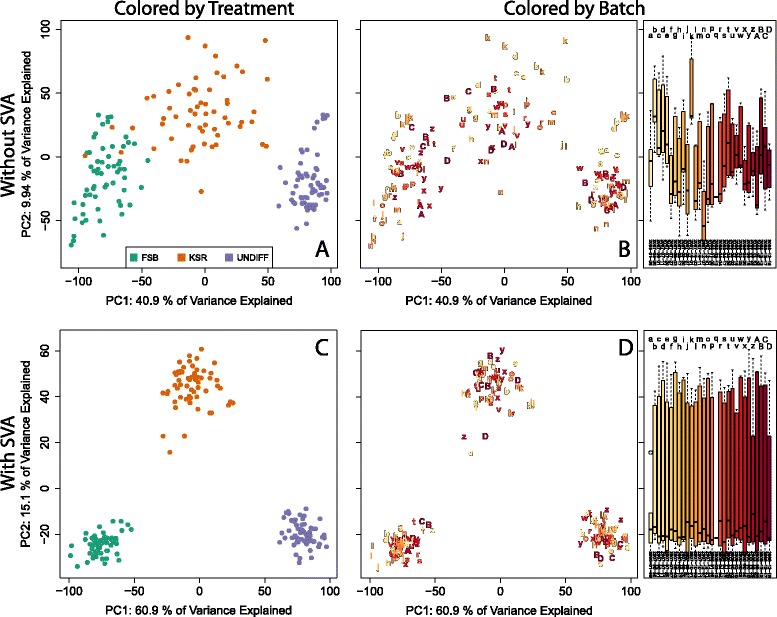
Global transcriptional landscape in differentiating pluripotent cells. PCA of expression data from differentiating pluripotent cells prior to SVA colored by differentiation treatment (**a**) and microarray scan date (**b**, *left panel*). The first PC shows a strong effect of treatment, while the second PC is related to “batch”: boxplots of the second PC indicate strong association with scan date (**b**, *right panel*). PCA following estimation and removal of SVs again colored by differentiation treatment (**c**) and microarray scan date (**d**, *left panel*). Both the first and second PC now show systematic association with differentiation, and the second PC no longer shows systematic change with scan date (**d**, *right panel*). Letters are also used to distinguish individual scan dates in (**b**) and (**d**)

Following normalization and preceding any “cleaning” of the data principal component analysis (PCA) is useful to gain a global perspective on the structure of the data (Fig. 1). While the first principal component (PC) reflects the differences between treatments (Fig. 1a), the second PC correlates strongly with processing data (Fig. 1b). This is common in gene expression data [11, 12].

To perform SVA here, we define the biological model as the average change in gene expression associated with each treatment (model coded in R as: “expression ~ treatment”, where treatment has 3 levels: undifferentiated, mesendoderm or neurectoderm). Hence, SVA will preserve this mean effect while removing other systematic heterogeneity in the data that effect many measures uncorrelated with this mean effect. After performing SVA and subsequently regressing out the 27 automatically determined SVs in this dataset, biological patterns are clearer in this global expression landscape (Fig. 1c). Now, the 1st PC constructs an axis of differentiation primarily towards a mesendodermal fate (it is of interest that the neuroectodermal fate also moves through this dimension). The 2nd PC identifies an axis that is exclusively represented in neuroectodermal differentiation. Importantly, these samples no longer cluster by processing date, indicating that SVA has successfully accounted for the batch effects while preserving biological signal (Fig. 1d). When combined with precise biological and technical data, these dimension-reducing plots provide a starting point for evaluating both global patterns of biology across the samples under study as well as the extent of technical variation in the data.

**Fig. 2:**
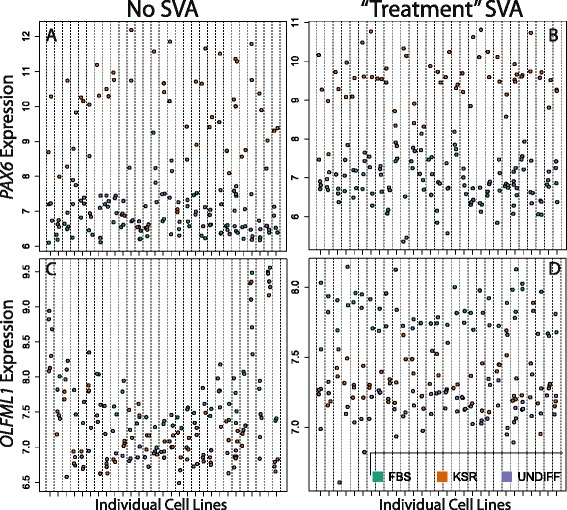
SVA improves power to identify differentially expressed genes. **a** *PAX6* shows significant differential expression between mesendodermal and neurectodermal differentiation before SVA (*p* = 1.77 × 10^−33^) and **b** this effect becomes more significant following SVA (*p* = 4.82 × 10^−53^). **c** Prior to SVA, *OLFML1* is not identified as being differentially expressed between differentiation conditions (*p* = 8.23 × 10^−5^), but **d** is highly significant after properly controlling for unwanted latent heterogeneity with SVA (*p* = 4.49 × 10^−29^). Expression values are on the log_2_ scale. Statistical significance was derived from a moderated t-statistic comparing expression in the mesendodermal differentiation condition versus that in the neurectodermal differentiation while also allowing variability to be explained by the undifferentiated condition (e.g. condition was categorical with 3 groups). Individual cell lines are represented on the X-axis. Gene expression on the Y-axis is depicted in quantile normalized, log2-scale intensities

We now turn from the impact of this “cleaning” procedure on global gene expression patterns to individual genes. We compared the results of gene-level contrasts between neuroectodermal and mesendodermal differentiation with and without SVA (Fig. 2 and Additional file 2: Figure S1). Genes expected to be differentially expressed by these treatments, such as *PAX6* [13], are significant without, but much more significant with SVA (Fig. 2a and b). We also highlight one gene, *OLFML1*, that has not previously been associated with neuroectodermal differentiation and which is non-significant before SVA (Fig. 2c) but very significant after (Fig. 2d). The majority of contrasts between neuroectodermal and mesendodermal differentiation are more significant after applying SVA (Additional file 2: Figure S1). Here, incorporating SVs as adjustment variables in individual-gene models reduces within treatment variability by assigning unrelated variance to the identified latent heterogeneity, hence increasing the power to identify biological differences. Furthermore, the relative ranks of genes previously reported to be involved with neuronal (Additional file 3: Figure S2A) and mesendodermal (Additional file 3: Figure S2B) differentiation are relatively preserved before and after SVA, demonstrating the relative stability of likely true positive effects.

It is crucial to note here that by defining the biological effect of interest to be the average change in expression with treatment, SVA removes many individual sample-specific expression traits. For example, there are multiple cell lines which do not induce *PAX6* expression in the neuroectodermal differentiation condition (Fig. 2a), but this is not observable after SVA (Fig. 2b). This is desirable if the goal is to estimate the mean effect of each treatment, but detrimental if expression phenotypes of individual cell lines are of interest (see discussion for additional details).

We note that similar reductions in technical variability are seen with the “ComBat” empirical Bayes batch correction approach [4], which also can also utilize an explicit definition of biological enquiry (Additional file 4: Figure S3A and B). However, the empirical Bayes method places less emphasis on the biological model, mostly reducing global variation even without specifying a biological model (e.g. using an intercept-only model, Additional file 4: Figure S3C and D). On the gene-level, ComBat produced smaller *p*-values on average (65.8 and 72.7 % of probes had smaller *p*-values under ComBat compared to SVA for the neuroectodermal versus mesendodermal fates, and neuroectodermal versus the undifferentiated state, respectively).

### Batch correction may remove secondary effects of interest

In Figs. 1 and 2, our biological model included only a treatment effect. If we are interested in exploring additional effects, this initial model specification is not complete. Analysis of additional effects requires their explicit inclusion in the biological model along with the primary effect of interest. Statistical analysis can then be applied to the data while incorporating the latent factors identified by the SV analysis.

**Fig. 3:**
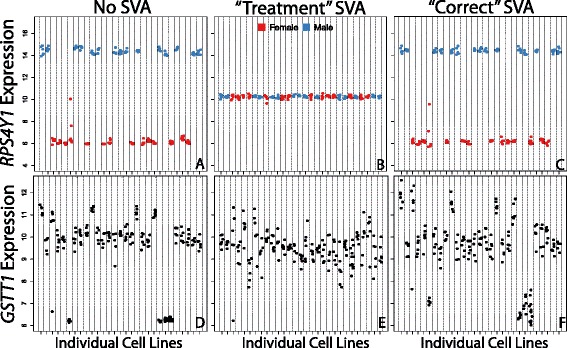
The biological model limits the scope of biological questions that can be asked. Defining a biological model that only preserves the effect of treatment obscures other true biological effects. The *RPS4Y1* gene is differentially expressed by sex (**a**). However, when the biological model passed to SVA does not include sex (i.e. using the treatment only model used in Figs. 1 and 2), the effect of sex at this gene is not apparent (**b**). When the effects defined in SVA include sex, the difference by sex is preserved in the data (**c**). Similarly, with *GSTT1*, copy number variation has a large impact on gene expression (**d**) which is removed by SVA under a treatment-only biological model (**e**). Including a term for *GSTT1* copy number in the biological model passed to SVA preserves the effect (**f**). Individual cell lines are represented on the X axis. Gene expression on the Y-axis is depicted in quantile normalized, log2-scale intensities

For example, if we are also interested in gene expression differences between the two sexes in the data depicted in Figs. 1 and 2, failure to include a “sex” variable in the biological model passed to SVA results in the removal of many sex effects in the data (Fig. 3a–c). Sex is correlated with the expression of multiple genes, and therefore appears as latent systematic heterogeneity to the SVA algorithm. The gene *RPS4Y1* (ribosomal protein S4, Y-linked 1) is on the Y-chromosome, and therefore only expressed in males. In the expression data analyzed without SVA, cell lines derived from males have over 256-fold increased expression of this gene (>8 units in the log2 scale; Fig. 3a). However, after removing the effects of the SVs derived from a treatment-only biological model (without including sex as a factor, i.e. using the biological model used to generate Figs. 1 and 2), this effect disappears entirely (Fig. 3b). Therefore, in order to preserve sex differences for exploration, a sex variable should be included along with treatment in the biological model for surrogate variable estimation (Fig. 3c; model coded in R as: “expression ~ treatment + sex”).

We explore another example at the *GSTT1* gene (glutathione S-transferase theta 1), which has common copy number variants (CNVs) in human populations [14] that are associated with *GSTT1* expression differences and local genetic structure of neighboring genes *GSTT2* and *GSTT2B* [15]. The expression data analyzed without SVA suggests three different copy numbers (0, 1 and 2) at this gene (Fig. 3d) which are validated with SNP microarray intensities in a subset of these samples (Additional file 5: Figure S4). Without accounting for *GSTT1* copy number (i.e. using the treatment-only biological model from Figs. 1 and 2) SVA removes the expression differences related to *GSTT1* CNV’s (Fig. 3e). However, accounting for copy number at this location in the biological model passed to SVA retains the copy number effect in the data (Fig. 3f; model coded in R as: “expression ~ treatment + CNV”).

Hence, when directed to preserve only the effect of treatment, SVA finds and removes wide-spread systematic expression effects associated with both sex and *GSTT1* copy number (among many other effects). This serves as a positive control indicating that SVA can successfully identify these known effects in expression data. This is also a cautionary note, reinforcing the notion that analyses must remain within the bounds set by the biological question defined at the outset of the data “cleaning”. Hence, any re-analyses of existing data with new questions requires an entirely novel re-processing of the data to address these new questions, not simply the calculation of additional statistics on the same processed data. Here we can clearly see how strong an impact our prior conception of the biological system can have on discovery in genomics data.

### Batch correction may remove non-linear expression patterns

Next, we re-analyzed an existing dataset measuring gene expression in the dorsolateral prefrontal cortex region (DLPFC) of the brain across the human lifetime. [16]. Data was processed and normalized as previously described [16]. Briefly, background correction and loess normalization were performed on raw two-channel intensity data, and low quality probes were removed from subsequent analysis, leaving 30,176 probes on 269 samples across the lifespan (fetal through the aged).

Patterns of gene expression across age are dynamic and non-linear: the largest changes occur during fetal life and infancy, and decrease in magnitude with age [16]. Here we expand on the previous modeling of age patterns across the lifespan in several key ways: 1) We applied splines to capture non-linear gene expression effects while ensuring patterns of gene expression are continuous across the lifespan The previous analysis used age by decade interaction terms, which are not necessarily continuous. 2) We estimated and adjusted for a much higher number of SVs. The previous analysis used only 2 SVs, here we allowed SVA to automatically determine this number: 31 SVs were used. This much increased “cleaning” further tuned this dataset to age effects (no doubt at the expense of many other effects such as those assessed in Fig. 3). Hence, this newly processed data should only be used for the estimation of canonical, mean patterns of expression across the lifetime. 3) We regressed out SVs while allowing the effects of age and mean gene expression (the intercept) to remain in the data. Previously, SVs were regressed out while ignoring possible correlation between SVs and age, potentially obscuring some age effects.

We applied three related spline models to the dataset, which were A) A linear spline with a knot at birth [2° of freedom], i.e. a line fit to expression across age in fetal life, and a second line fit to expression across age in postnatal life. B) A 2nd degree basis spline with a knot at birth [3° of freedom], i.e. a curve fit to expression across age in fetal life, and a second curve fit to expression across age in postnatal life. C) A 2nd degree basis spline with knots at birth, 1, 10, 20 and 50 years [8° of freedom], i.e. a curve fit to expression across age within each age range between these knots. Each model also allowed an offset at birth, because there were no samples in the third trimester of fetal life.

**Fig. 4:**
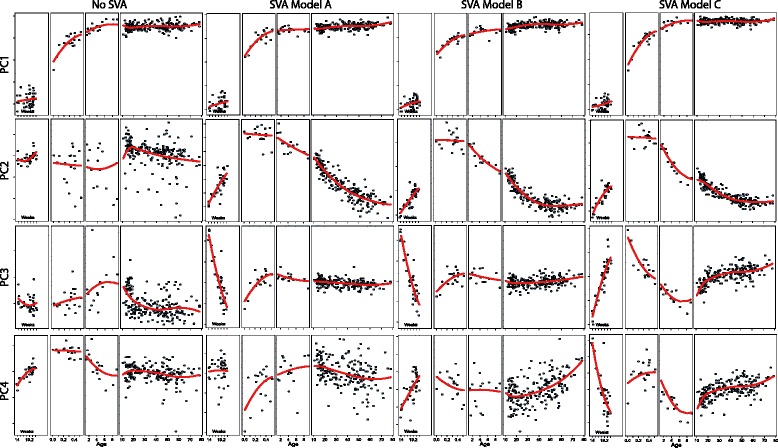
Global view of the impact of differing models in SVA. PCA performed on the original normalized data (“No SVA”), and on data “cleaned” with SVA using the 3 different models described in the text (**a**, **b** and **c**). Values of individual samples in the first 4 PCs are shown for each analysis, along with a smoothing spline to these PC’s across age (*red*). SVA Model A: linear spline with a knot at birth [2° of freedom], SVA Model B: 2nd degree basis spline with a knot at birth [3° of freedom], SVA Model C: 2nd degree basis spline with knots at birth, 1, 10, 20 and 50 years [8° of freedom]. Each model also incorporated an offset at birth

**Fig. 5:**
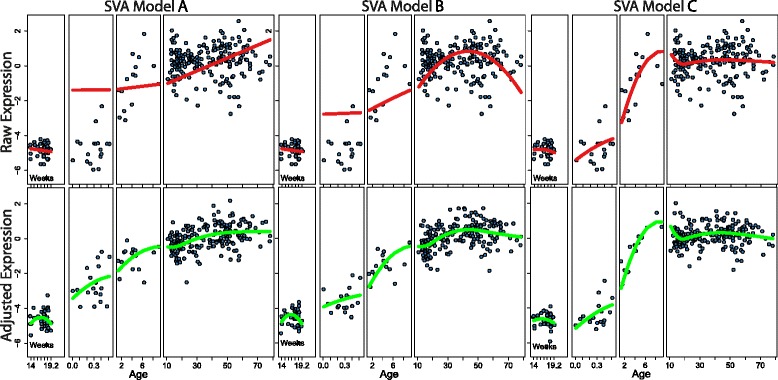
Individual gene view of the impact of differing models in SVA. CNDP1 gene expression is shown in the original normalized data (“Raw”) in the top panels. Each of the 3 different models used for SVA are shown overlaid on this original data in the top panels. This fit represents the effect that SVA will preserve for this particular gene in each of the 3 different scenarios (*red*). Bottom panels show CNDP1 gene expression adjusted with SVs generated using each of the 3 different models. Smoothing splines for expression across age are shown for each to depict the difference in expression patterns present when using SVA under the different models (*green*)

We applied SVA to the normalized raw data under each model described above, allowing each to have its own set of SVs, which were regressed out of the data to remove their effects for visualization. The impact of each different model employed with SVA is depicted globally using PCA (Fig. 4) and at the individual gene level (Fig. 5). Consistently, increasing model complexity and flexibility produces PC’s which are more dynamic across age with the least variance within age (variance within age in model A > B > C, and dynamics across age in model A < B < C; Fig. 4). These effects are increasingly clear in deeper PC’s. It is of particular interest that this increased power to identify global patterns in the data is achieved while maintaining higher fidelity to the original normalized data: In ~2/3 of the ~30 K probes measured, the adjustment to the data made by SVA under model C is less than that made by SVA under either of the other models (Additional file 6: Figure S5).

Inspection of the impact of these different models on SVA’s adjustment of individual gene data demonstrates that increased flexibility in modeling effects can result in both higher fidelity to the original data and the increased ability to distinguish specific dynamic patterns (Fig. 5): Models A and B introduce greater adjustments to the original normalized data than model C. Model C, however effectively reduces within age variance while preserving a gene expression pattern apparent in the normalized data in which dynamics are limited to the range of birth-10 year. While perhaps reducing some variance, both models A and B produce a pattern of expression across age where dynamics are spread from birth-50 year, demonstrating the increased resolution of pattern identification possible with the more flexible model C. This newly processed data is available along with original raw data at the GEO repository: GSE30272.

## Conclusions

Using SVA, ComBat or related tools that require the precise definition of biological enquiry can increase the power to identify specific signals in complex genomic datasets. However, especially when high levels of correction are used (e.g. when the automatic number of SVs generated by SVA is used), biological discovery is directly limited by researchers’ prior conception of the system under study. Hence, we must be precise and deliberate in the design and analysis of experiments and the resulting data, and also mindful of the limitations we impose with our own perspective.

Here we have discussed primarily how to focus as much as possible on a single narrowly defined question. Methods such as SVA can also be used in the more open exploration of genomics data. For this, the definition of the biological model of interest would have to be designed specifically for flexibility, to allow more diverse effects to coexist in the SV-adjusted data. Perhaps equally important would be the reduction of the amount of “cleaning” performed. In the use of SVA this would be done by tempering the number of SVs identified and used for the adjustment of the data so that large components of unwanted heterogeneity can be removed while leaving intact a broad range of biological effects to explore. We do note that performing SVA does not necessarily produce overly optimistic results, as the SVA algorithm allows for potential correlation between the primary variables and estimated latent variables. Regardless, this is a balancing act that necessarily entails iterative data processing and assessment of global and gene-specific impacts of the analysis, and can be guided by perspectives and plots that we illustrate in this report.

While we have primarily explored the SVA batch correction algorithm, we note comparable global and gene-level results using the ComBat batch-correction algorithm [4] within the stem cell dataset. However, as the ComBat algorithm requires categorical “batch” variables, we could not optimally implement it in the brain dataset - several of the top principal components associated with the quantitative RNA integrity numbers (RIN), suggesting that tissue quality likely induces “batch”-like effects in the data. Similarly, while we view the Remove Unwanted Variation (RUV) algorithm [6] a natural extension of the SVA algorithm, we found that many housekeeping genes [17], typically used as “negative control genes” in the algorithm, were differentially expressed by differentiation in the stem cell dataset (Additional file 7: Figure S6) and development/birth in the brain dataset, highlighting the difficulty in selecting an a priori set of negative controls required by the algorithm. The selection of “negative control genes” that associate with the outcome of interest has a similar effect as SVA model misspecification displayed in Fig. 3. Without studies dedicated to the identification of expressed yet outcome-independent genes within the system under study, the selection of such control genes is quite difficult, although recent work has suggested that the approach may be robust to the specific choice of control genes [18], particularly when technical replicate samples have been generated [19].

The analysis structures described here focus on mean effects across all samples studied. Analysis of experiments in dynamic systems including replicates of a wide diversity of individual subjects and well-characterized genomes will be necessary to move beyond the study of average population effects, into functional genomics where we may begin to estimate the impact of individual genomes on precise molecular and cellular phenotypes. In this context, the use of data “cleaning” must be used with extreme caution as it can remove a great deal of information from genomic data as we have demonstrated here.

### Code

All code for processing, analyzing and visualizing is available at: https://github.com/andrewejaffe/StemCellSVA.

## Additional files

Additional file 1: Supplementary Methods and Supplementary Figure Legends. (DOCX 19 kb) Additional file 2: Figure S1. Transcriptome-wide changes in differential expression from SVA. (A) Log fold change for neurectodermal (KSR) versus mesendodermal (FBS) treatment effects before (y-axis) and after (x-axis) SVA. (B) Variance in regression model for treatment effect before and after SVA. (C) –log_10_ *p*-values from moderated t-statistics calculated using the log fold changes in (A). Dot color in each panel identifies genes depicted in Fig. 2 – *PAX6* is blue and *OLFML1* is red. (PDF 2226 kb) Additional file 3: Figure S2. Gene ranks before and after SVA. Ranks of genes (by *p*-value) for (A) mesendodermal and (B) neuroectoderal differentiation conditions before and after SVA. Highlighted genes are (A) *MBNL2, ZHX1, TOM1L2, HGSNAT, SSFA2, FKBP9, PARVA, LRRC8B, NPM3, SNURF, RTN3, EIF5A2, CUTC, SALL2* and (B) *TSPAN31, BBS10, TARBP1,C17orf69, ABI2, NRCAM, SCG5, SMAD7, CORO2A, SLC25A24* which have been previously implicated in each differentiation condition. (PDF 1764 kb) Additional file 4: Figure S3. Global transcriptional landscape in differentiating pluripotent cells using ComBat [4]. PCA of expression data from differentiating pluripotent cells prior to ComBat colored by differentiation treatment (A) and microarray scan date (B, left panel). The first PC shows a strong effect of treatment, while the second PC is related to “batch”: boxplots of the second PC indicate strong association with scan date (B, right panel). PCA following shrinkage again colored by differentiation treatment (C) and microarray scan date (D, left panel). Both the first and second PC now show systematic association with differentiation, and the second PC no longer shows systematic change with scan date (D, right panel). Letters are also used to distinguish individual scan dates in (B) and (D). (PDF 304 kb) Additional file 5: Figure S4. Raw copy number estimates via microarray intensities. (A) log R ratios and (B) B-allele frequencies from Illumina SNP microarrays for the various cell lines in the expression dataset. A log R ratio of 0 indicates 2 copies of the gene, which also corresponds to B-allele frequencies near 0, 0.5, and/or 1. (PDF 297 kb) Additional file 6: Figure S5. Model comparison density plots. (A) SVA Model C versus Model A, (B) SVA Model C versus Model B, and (C) SVA Model B versus Model A. Each panel contains one point per probe, and groups of nearby points are shaded by density. Note that each model is relative to a model of no SVA, so that that Y-axis: log_10_(Σ(Model 1 – None)^2^) – log_10_(Σ(Model 2 – None)^2^) and X-axis: (log_10_(Σ(Model 1 – None)^2^) + log_10_(Σ(Model 2 – None)^2^))/2 which roughly translate into the typical “MA” plot in the microarray literature [20]. The dashed black line is 0 and the solid red line corresponds to the mean difference in model fits. Numbers indicate how many points are above and below 0. (PDF 1057 kb) Additional file 7: Figure S6. Distribution of differential expression signal at housekeeping genes. Housekeeping genes (solid lines) have very significant T-statistics for differentiation conditions suggesting they would be poor “control” genes for an algorithm like Remove Unwanted Variation (RUV) [6]. (PDF 18 kb)
