# Erratum to: Practical impacts of genomic data “cleaning” on biological discovery using surrogate variable analysis

**DOI:** 10.1186/s12859-016-1152-0

**Published:** 2016-08-10

**Authors:** Andrew E. Jaffe, Thomas Hyde, Joel Kleinman, Daniel R. Weinberger, Joshua G. Chenoweth, Ronald D. McKay, Jeffrey T. Leek, Carlo Colantuoni

**Affiliations:** 1 Lieber Institute for Brain Development, 855 N Wolfe St, Ste 300, Baltimore, MD 21205 USA; 2 Department of Biostatistics, Johns Hopkins Bloomberg School of Public Health, 615 N Wolfe St, Baltimore, MD 21205 USA; 3 Department of Neurology, Johns Hopkins School of Medicine, Baltimore, MD 21205 USA; 4 Department of Psychiatry, Johns Hopkins School of Medicine, Baltimor, MD 21205 USA; 5 Department of Neuroscience, Johns Hopkins School of Medicine, Baltimore, Maryland 21205 USA; 6 McKusick-Nathans Institute of Genetic Medicine, Johns Hopkins School of Medicine, Baltimore, Maryland 21205 USA

After publication of the original article [1] it was brought to our attention that author Daniel R. Weinberger was incorrectly included as Daniel R. Weinbergern. The correct spelling of the name is included in the author list of this erratum.
