# Supplementary material for: Practical impacts of genomic data “cleaning” on biological discovery using surrogate variable analysis
Source: BMC Bioinformatics. 2015 Nov 6;16:372. doi: 10.1186/s12859-015-0808-5 (PMC4636836; doi:10.1186/s12859-015-0808-5)
Supplement: Additional file 1: — Supplementary Methods and Supplementary Figure Legends. (DOCX 19 kb) [file 12859_2015_808_MOESM1_ESM.docx]

**Additional file 1**

**Practical Impacts of Genomic Data “Cleaning” on Biological Discovery using Surrogate Variable Analysis**

**By: Andrew Jaffe et al.**

**Supplementary Methods**

Stem Cell Data:

Single channel Agilent G4112F microarray data from StemCellDB ([Mallon et al, 2012](#_ENREF_5)) was obtained from the Gene Expression Omnibus ([Edgar et al, 2002](#_ENREF_1)). Processing date was extracted from the header of each array. The raw microarray data was preprocessed and normalized using the ‘limma’ Bioconductor package ([Smyth, 2005](#_ENREF_6)), consisting of background subtract (offset = 50) and quantile normalization across all samples. Surrogate variables (SVs) were estimated with the ‘sva’ Bioconductor package ([Leek et al, 2012](#_ENREF_4)). The sex of each cell line was estimated from the *DDX3Y* gene on the Y chromosome. Linear models were implemented using ‘limma’ package ([Smyth, 2005](#_ENREF_6)), and SVs were included as adjustment variables in subsequent models. R code is available at <https://github.com/andrewejaffe/StemCellSVA>

Brain Data:

All code is available at: <https://github.com/andrewejaffe/StemCellSVA>

**References**

Edgar R, Domrachev M, Lash AE (2002) Gene Expression Omnibus: NCBI gene expression and hybridization array data repository. *Nucleic acids research* **30:** 207-210

Gagnon-Bartsch JA, Speed TP (2012) Using control genes to correct for unwanted variation in microarray data. *Biostatistics* **13:** 539-552

Johnson WE, Li C, Rabinovic A (2007) Adjusting batch effects in microarray expression data using empirical Bayes methods. *Biostatistics* **8:** 118-127

Leek JT, Johnson WE, Parker HS, Jaffe AE, Storey JD (2012) The sva package for removing batch effects and other unwanted variation in high-throughput experiments. *Bioinformatics* **28:** 882-883

Mallon BS, Chenoweth JG, Johnson KR, Hamilton RS, Tesar PJ, Yavatkar AS, Tyson LJ, Park K, Chen KG, Fann YC, McKay RDG (2012) StemCellDB: The Human Pluripotent Stem Cell Database at the National Institutes of Health. *Stem Cell Research*

Smyth GK (2005) Limma: linear models for microarray data. In *Bioinformatics and Computational Biology Solutions using R and Bioconductor*, Gentleman R, Carey V, Dudoit S, Irizarry R, Huber W (eds), pp 397-420. Springer, New York

Yang YH, Dudoit S, Luu P, Lin DM, Peng V, Ngai J, Speed TP (2002) Normalization for cDNA microarray data: a robust composite method addressing single and multiple slide systematic variation. *Nucleic acids research* **30:** e15
