# Supplementary figures and images for: Practical impacts of genomic data “cleaning” on biological discovery using surrogate variable analysis
Source: BMC Bioinformatics. 2015 Nov 6;16:372. doi: 10.1186/s12859-015-0808-5 (PMC4636836; doi:10.1186/s12859-015-0808-5)

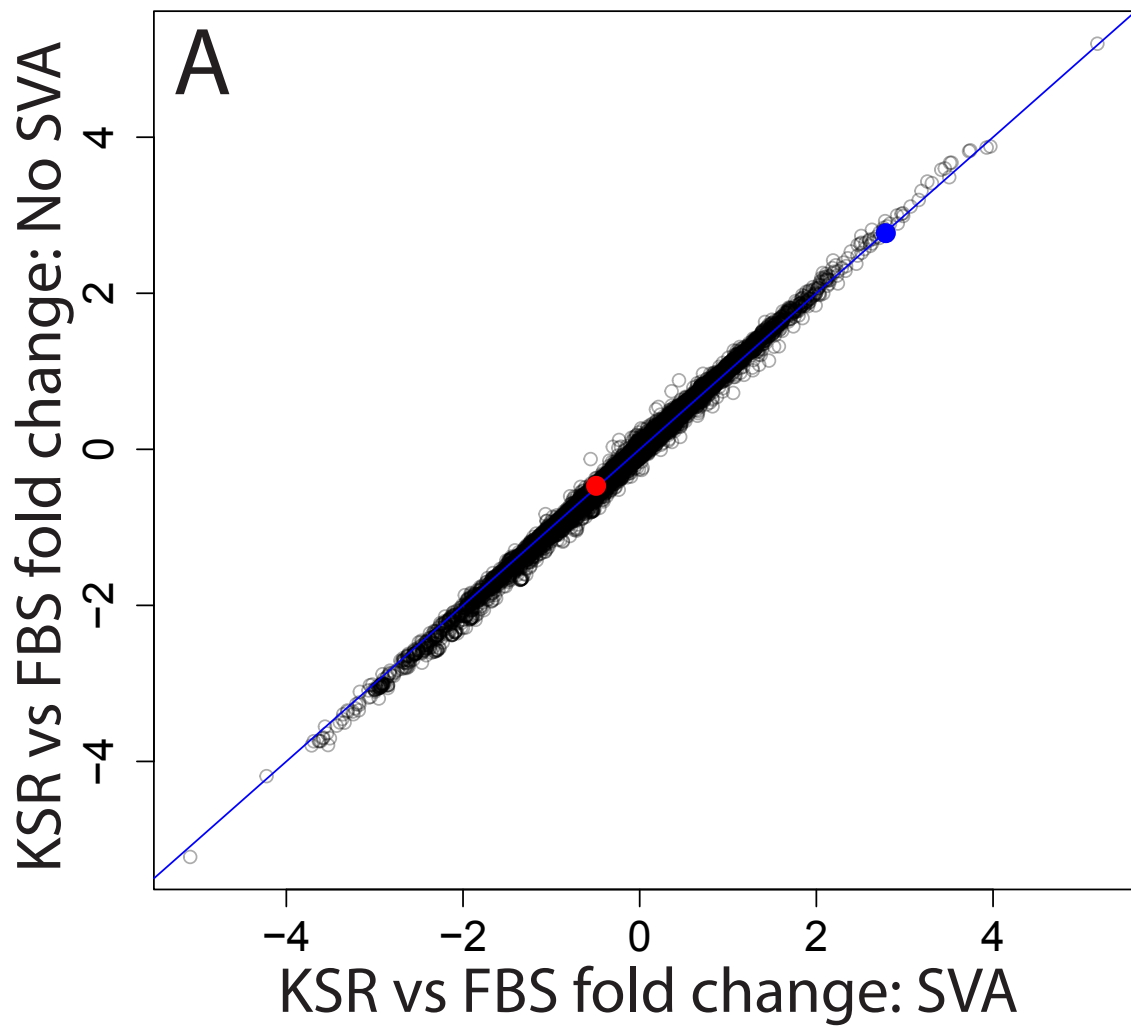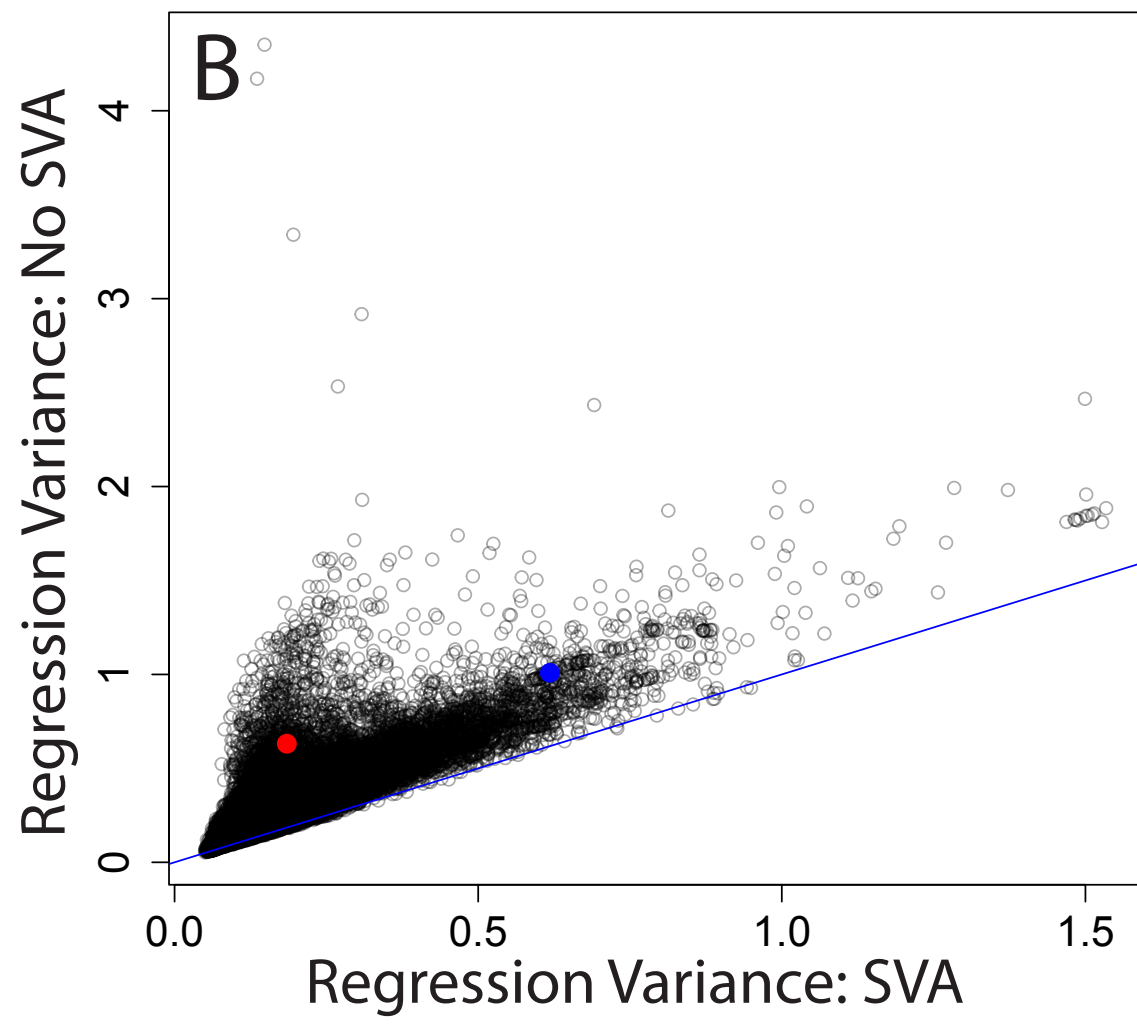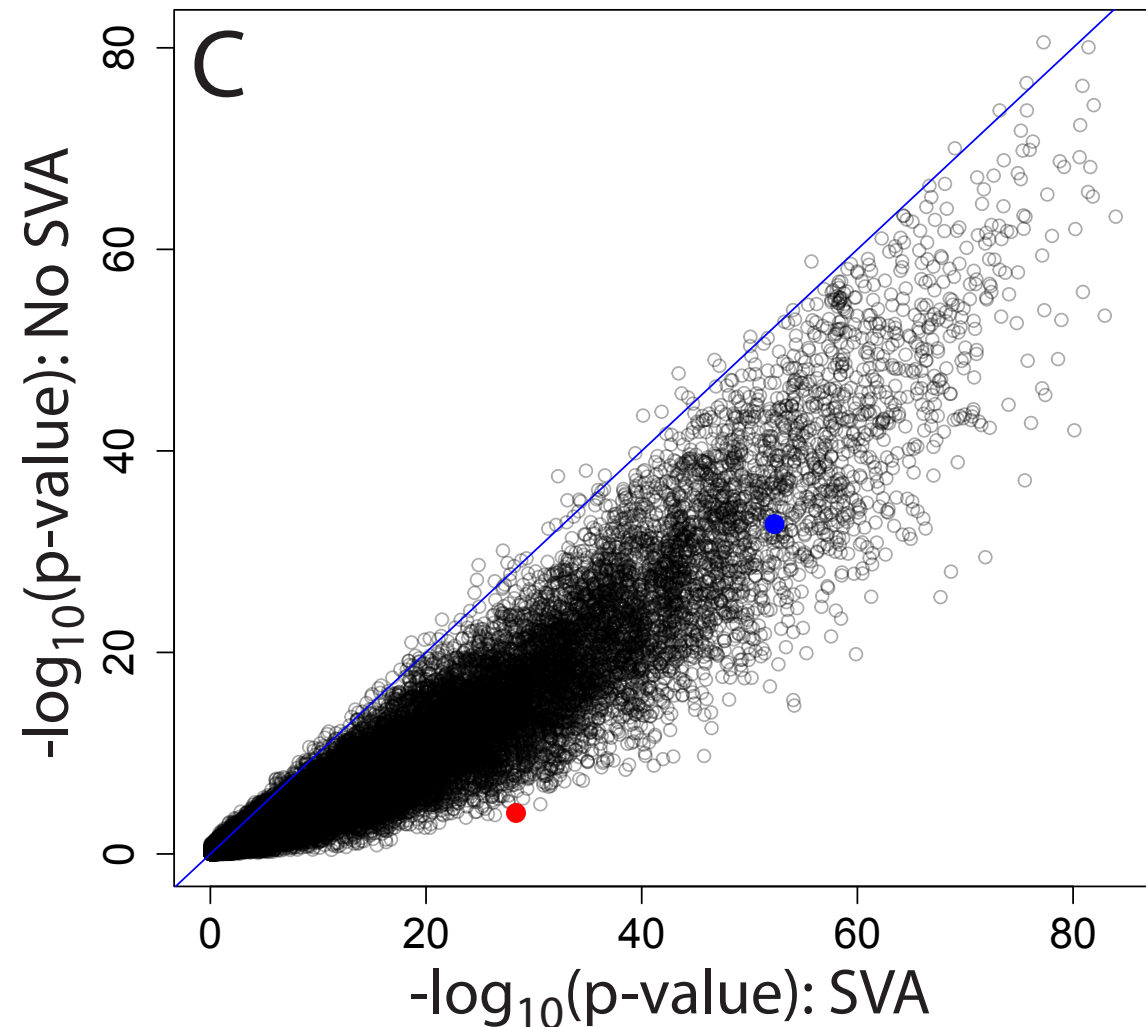

Supplement: Additional file 2: Figure S1. — Transcriptome-wide changes in differential expression from SVA. (A) Log fold change for neurectodermal (KSR) versus mesendodermal (FBS) treatment effects before (y-axis) and after (x-axis) SVA. (B) Variance in regression model for treatment effect before and after SVA. (C) –log10 p-values from moderated t-statistics calculated using the log fold changes in (A). Dot color in each panel identifies genes depicted in Fig. 2 – PAX6 is blue and OLFML1 is red. (PDF 2226 kb) [file 12859_2015_808_MOESM2_ESM.pdf]

# Mesendodermal

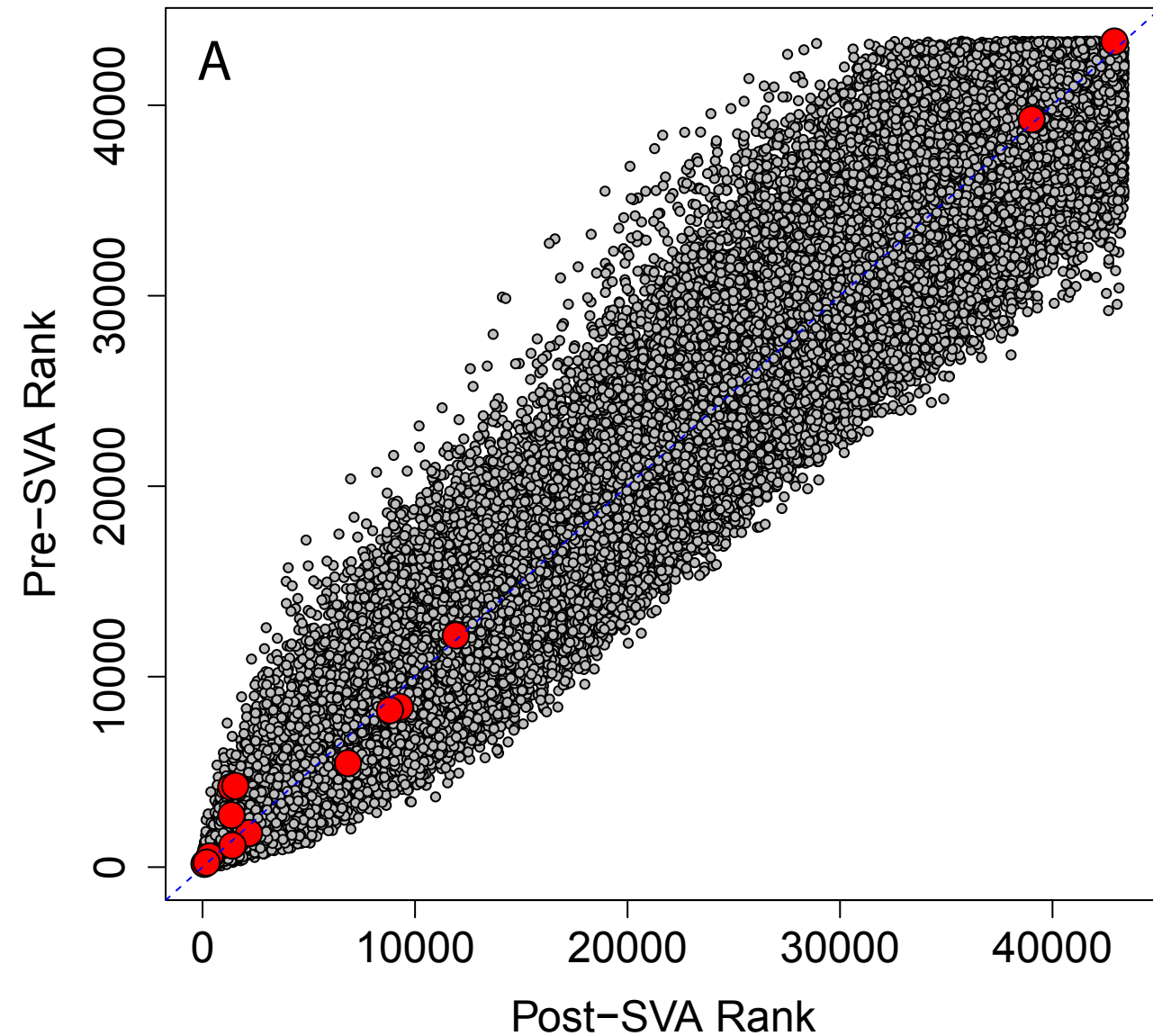

# Neuroectodermal

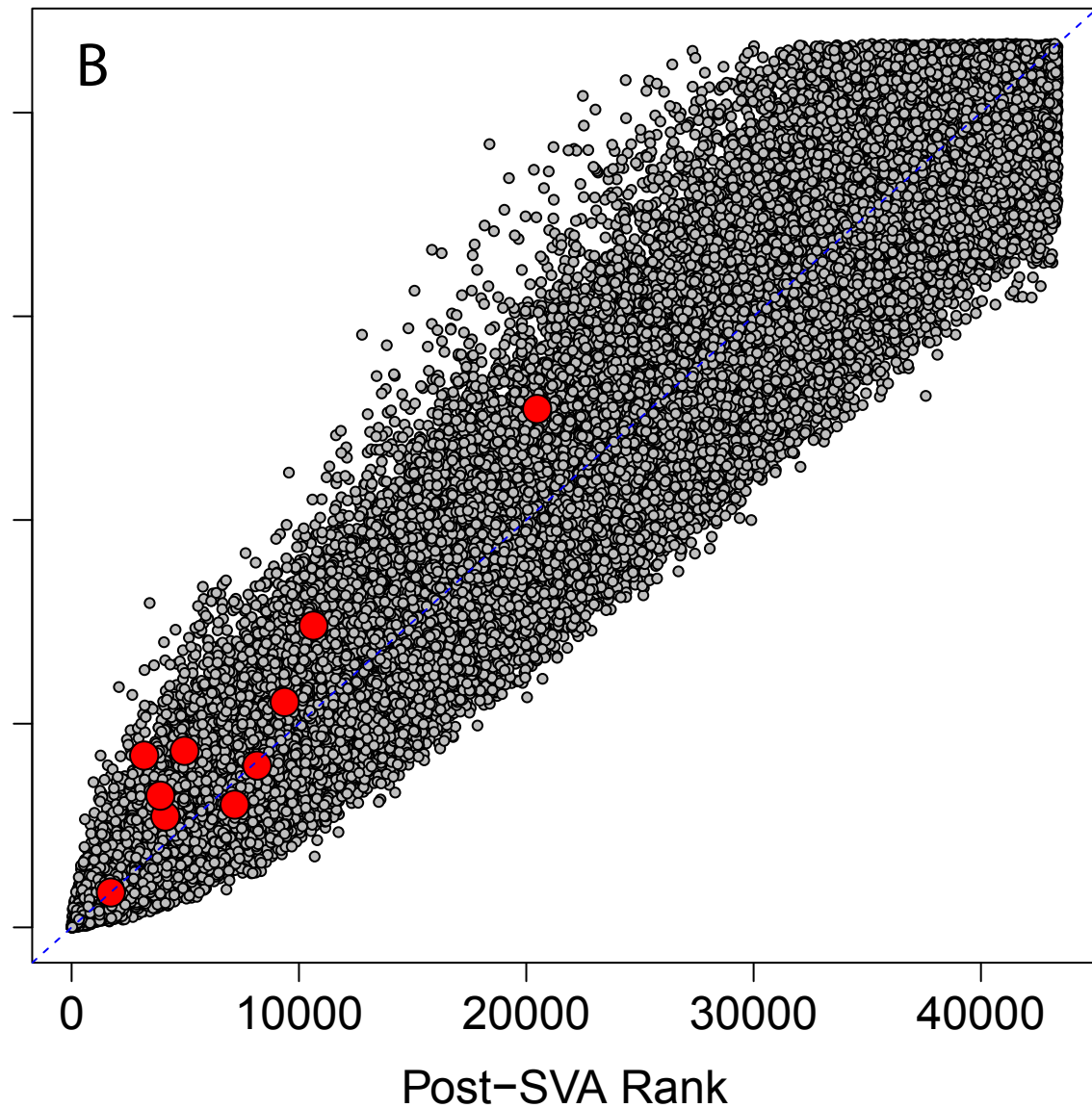

Supplement: Additional file 3: Figure S2. — Gene ranks before and after SVA. Ranks of genes (by p-value) for (A) mesendodermal and (B) neuroectoderal differentiation conditions before and after SVA. Highlighted genes are (A) MBNL2, ZHX1, TOM1L2, HGSNAT, SSFA2, FKBP9, PARVA, LRRC8B, NPM3, SNURF, RTN3, EIF5A2, CUTC, SALL2 and (B) TSPAN31, BBS10, TARBP1,C17orf69, ABI2, NRCAM, SCG5, SMAD7, CORO2A, SLC25A24 which have been previously implicated in each differentiation condition. (PDF 1764 kb) [file 12859_2015_808_MOESM3_ESM.pdf]

SVA Model "C" Versus "A"

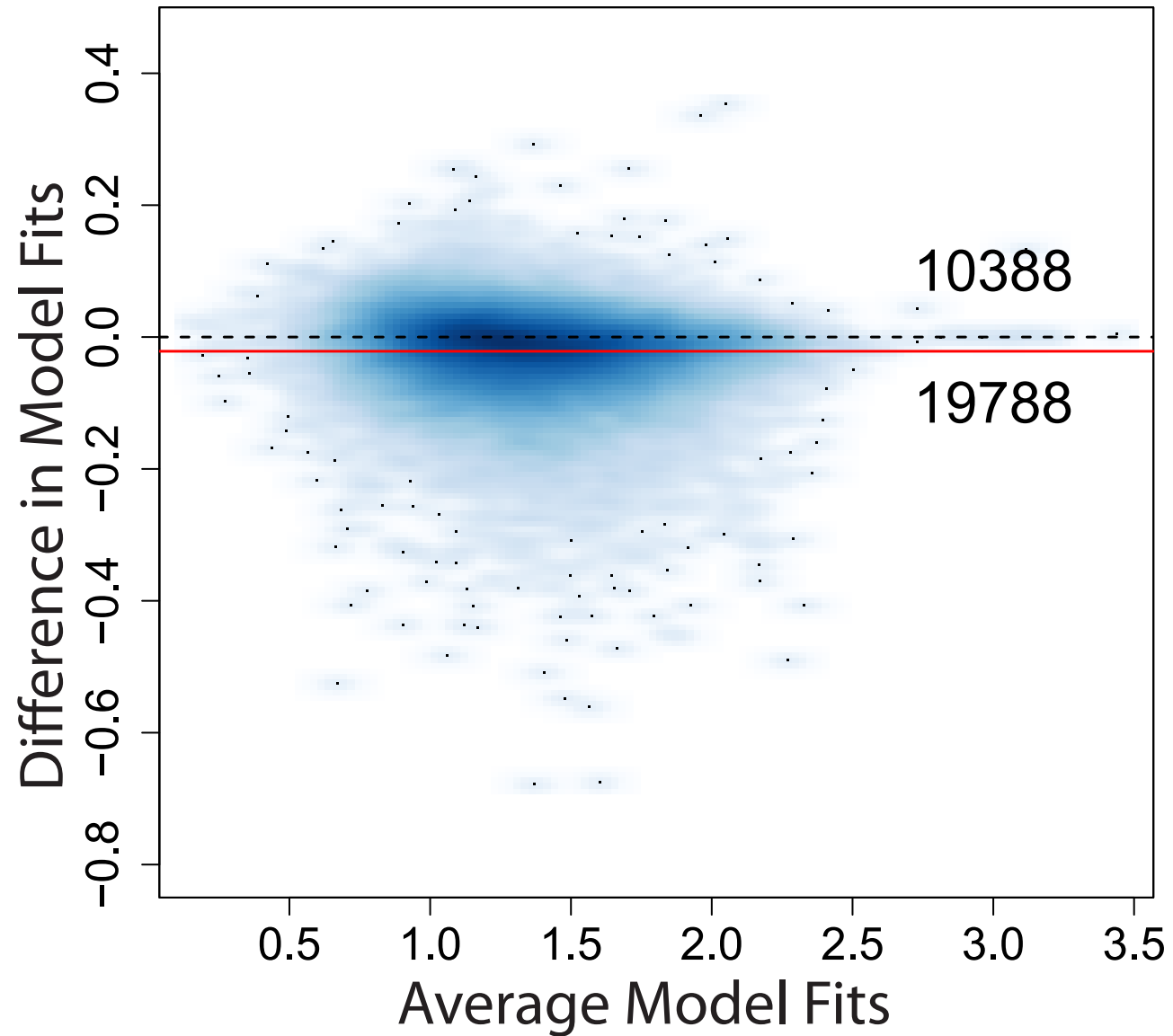

SVA Model "C" Versus "B"

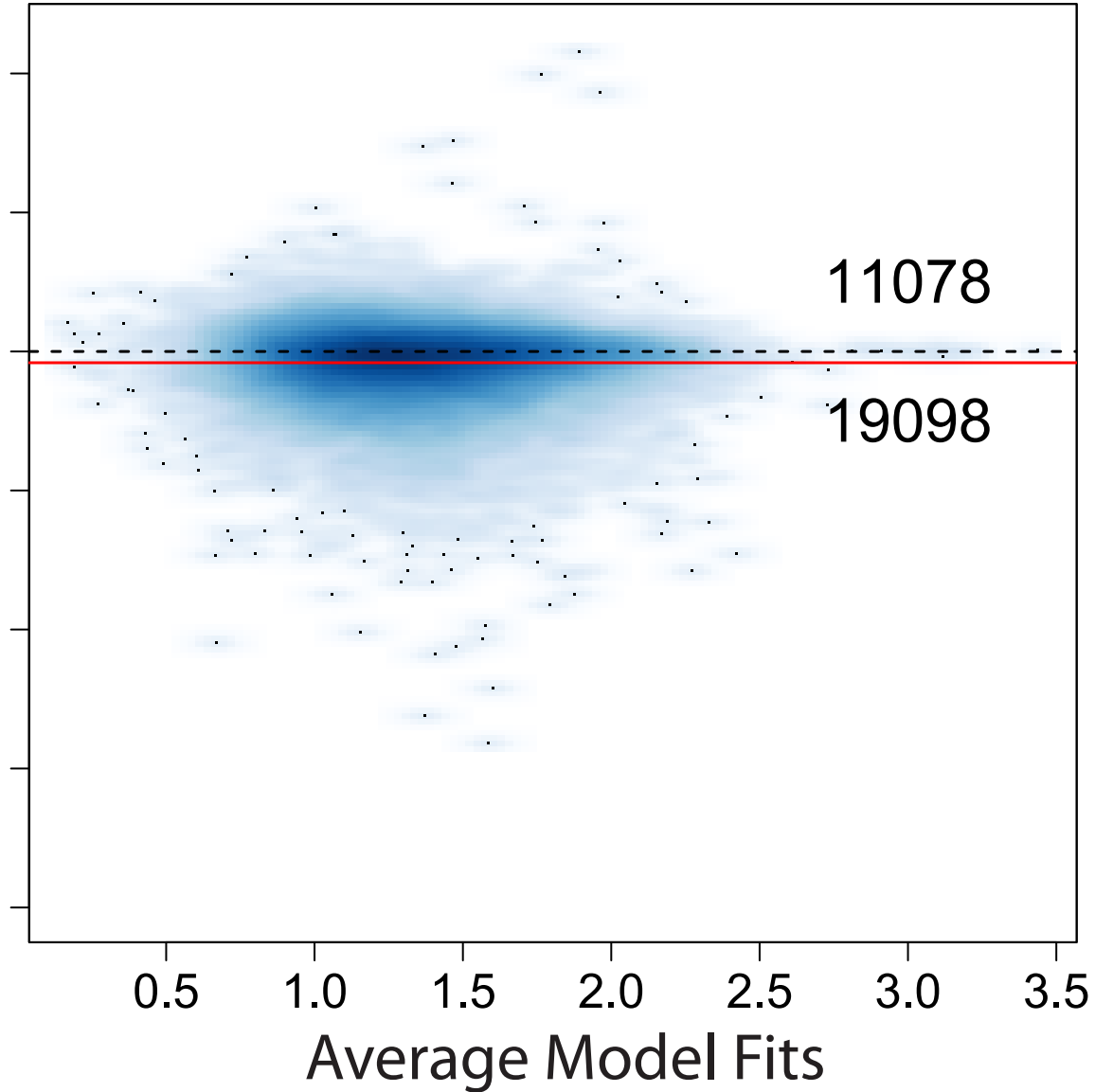

SVA Model "B" Versus "A"

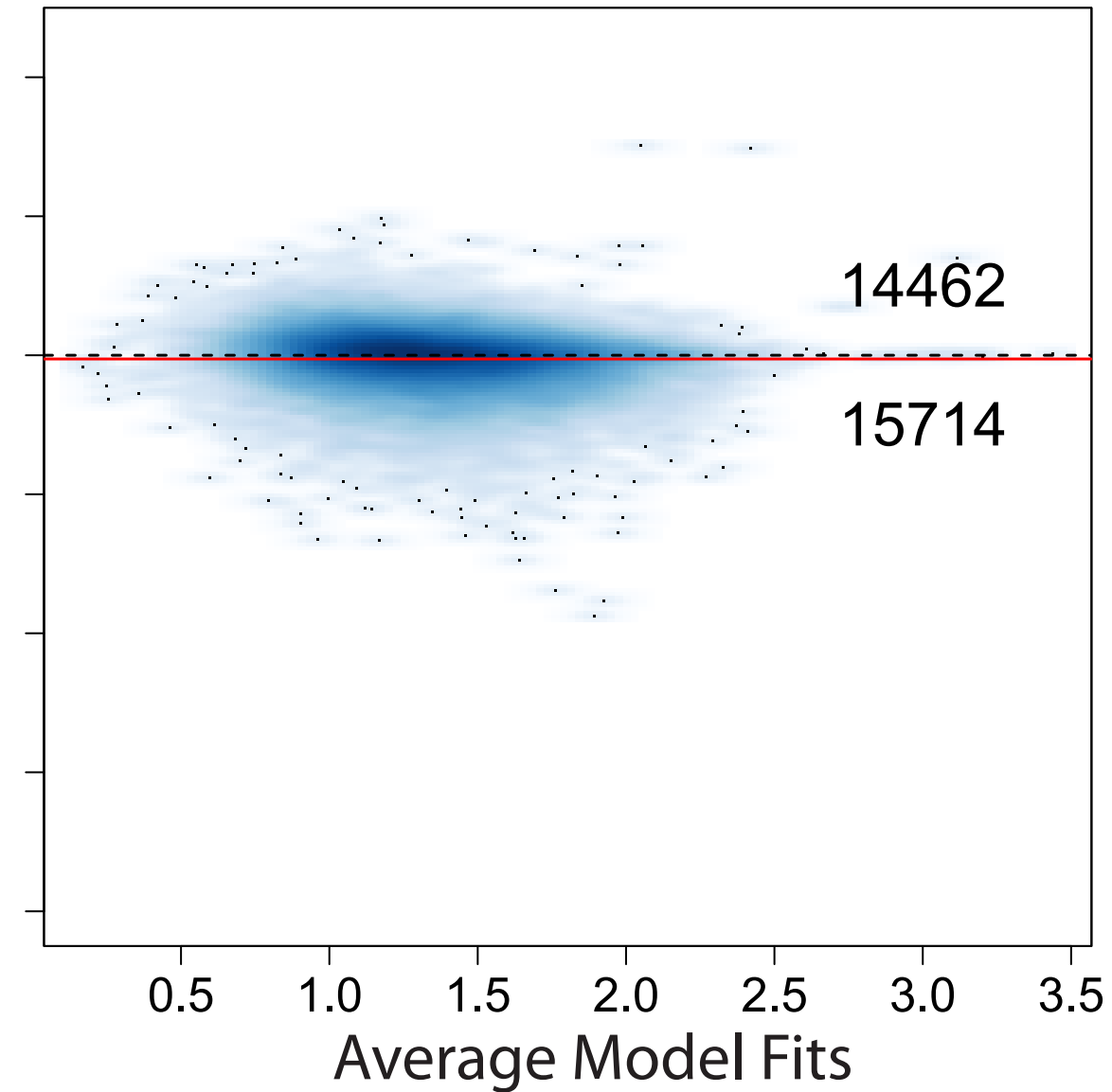

Supplement: Additional file 6: Figure S5. — Model comparison density plots. (A) SVA Model C versus Model A, (B) SVA Model C versus Model B, and (C) SVA Model B versus Model A. Each panel contains one point per probe, and groups of nearby points are shaded by density. Note that each model is relative to a model of no SVA, so that that Y-axis: log10(Σ(Model 1 – None)2) – log10(Σ(Model 2 – None)2) and X-axis: (log10(Σ(Model 1 – None)2) + log10(Σ(Model 2 – None)2))/2 which roughly translate into the typical “MA” plot in the microarray literature [20]. The dashed black line is 0 and the solid red line corresponds to the mean difference in model fits. Numbers indicate how many points are above and below 0. (PDF 1057 kb) [file 12859_2015_808_MOESM6_ESM.pdf]

# Housekeeping Gene T-stat Distribution

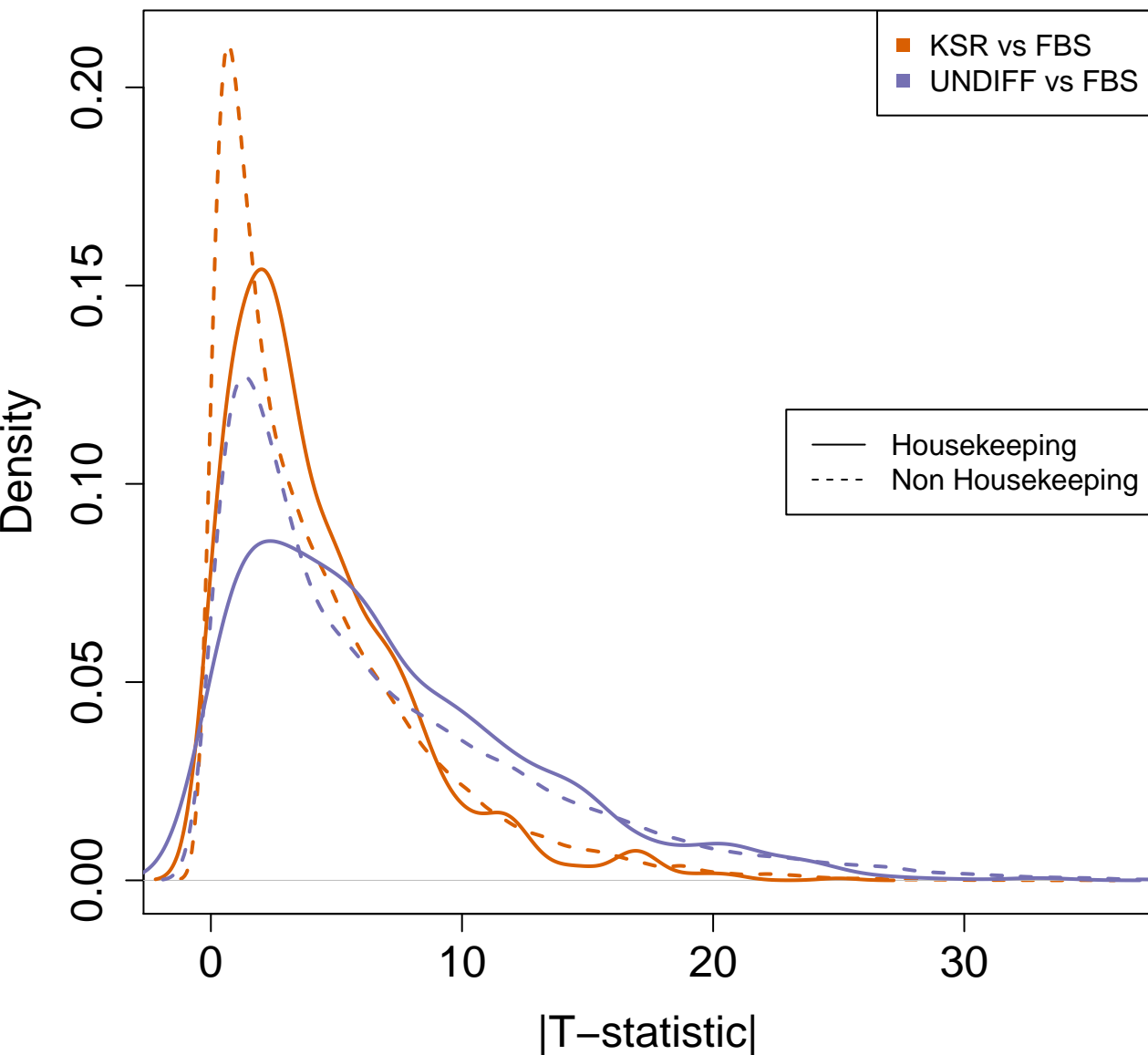

Supplement: Additional file 7: Figure S6. — Distribution of differential expression signal at housekeeping genes. Housekeeping genes (solid lines) have very significant T-statistics for differentiation conditions suggesting they would be poor “control” genes for an algorithm like Remove Unwanted Variation (RUV) [6]. (PDF 18 kb) [file 12859_2015_808_MOESM7_ESM.pdf]
