# Supplementary material for: Practical impacts of genomic data “cleaning” on biological discovery using surrogate variable analysis
Source: BMC Bioinformatics. 2015 Nov 6;16:372. doi: 10.1186/s12859-015-0808-5 (PMC4636836; doi:10.1186/s12859-015-0808-5)
Supplement: Additional file 4: Figure S3. — Global transcriptional landscape in differentiating pluripotent cells using ComBat [4]. PCA of expression data from differentiating pluripotent cells prior to ComBat colored by differentiation treatment (A) and microarray scan date (B, left panel). The first PC shows a strong effect of treatment, while the second PC is related to “batch”: boxplots of the second PC indicate strong association with scan date (B, right panel). PCA following shrinkage again colored by differentiation treatment (C) and microarray scan date (D, left panel). Both the first and second PC now show systematic association with differentiation, and the second PC no longer shows systematic change with scan date (D, right panel). Letters are also used to distinguish individual scan dates in (B) and (D). (PDF 304 kb) [file 12859_2015_808_MOESM4_ESM.pdf]

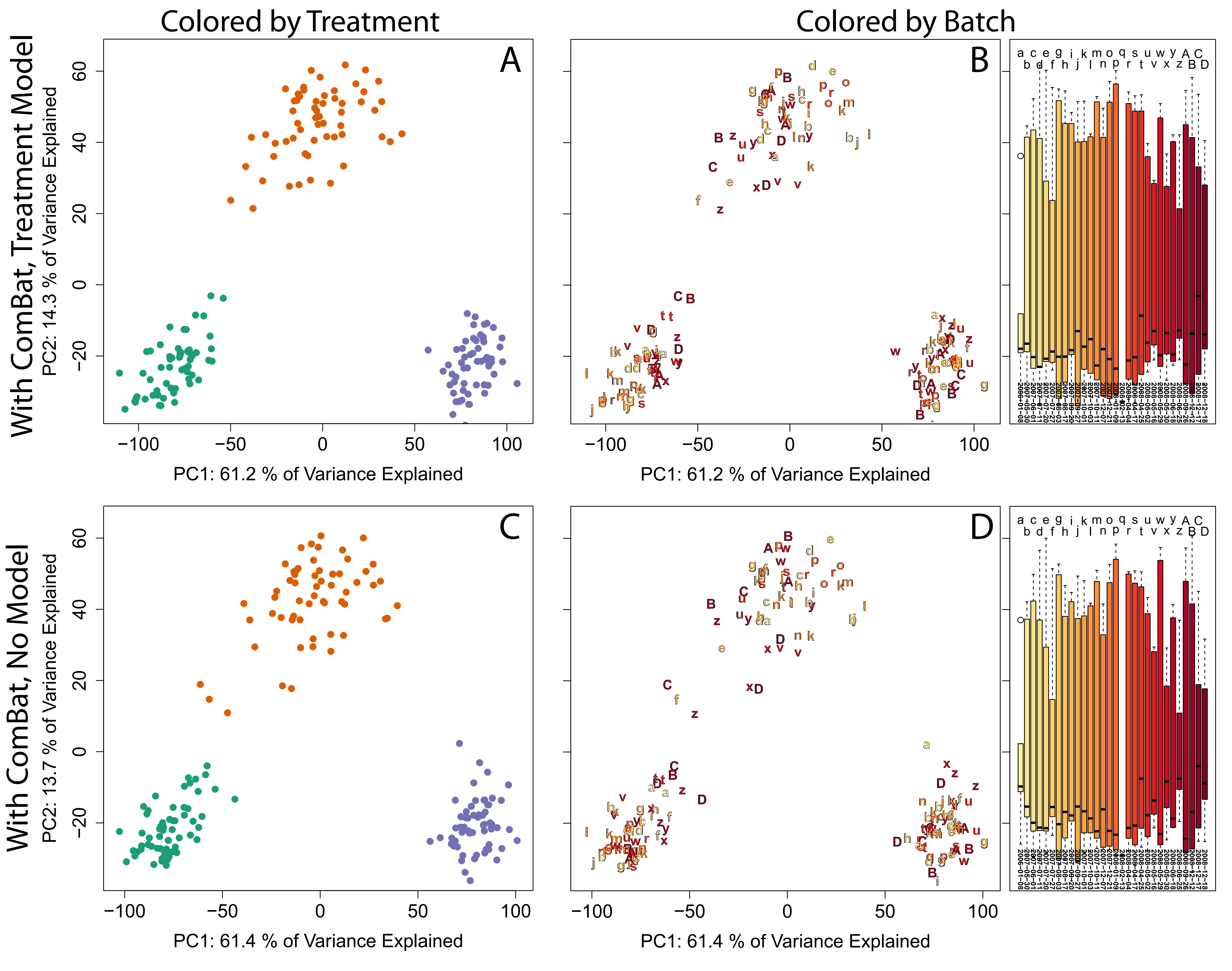

Colored by Treatment
Colored by Batch
With ComBat, Treatment Model
With ComBat, No Model
A
B
C
D
PC2: 14.3 % of Variance Explained
PC2: 13.7 % of Variance Explained
PC1: 61.2 % of Variance Explained
PC1: 61.2 % of Variance Explained
PC1: 61.4 % of Variance Explained
PC1: 61.4 % of Variance Explained
