# Supplementary material for: Practical impacts of genomic data “cleaning” on biological discovery using surrogate variable analysis
Source: BMC Bioinformatics. 2015 Nov 6;16:372. doi: 10.1186/s12859-015-0808-5 (PMC4636836; doi:10.1186/s12859-015-0808-5)
Supplement: Additional file 5: Figure S4. — Raw copy number estimates via microarray intensities. (A) log R ratios and (B) B-allele frequencies from Illumina SNP microarrays for the various cell lines in the expression dataset. A log R ratio of 0 indicates 2 copies of the gene, which also corresponds to B-allele frequencies near 0, 0.5, and/or 1. (PDF 297 kb) [file 12859_2015_808_MOESM5_ESM.pdf]

LogR: Resolution = 10000 bp

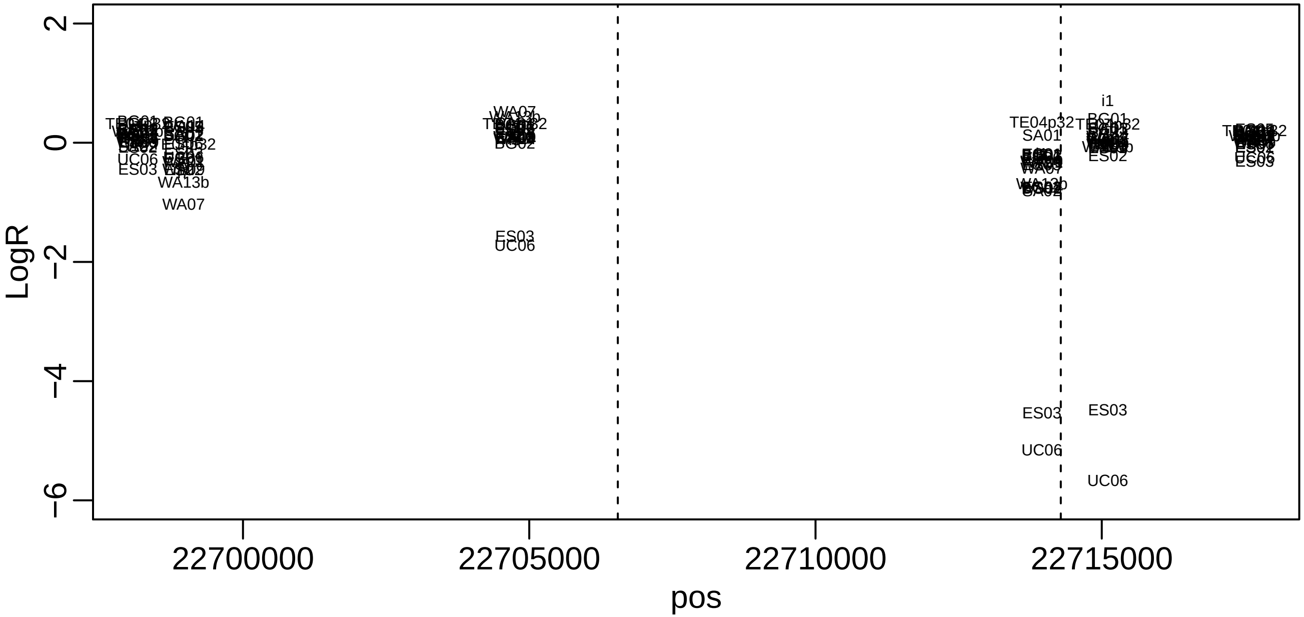

B Allele Freq: Resolution = 10000 bp

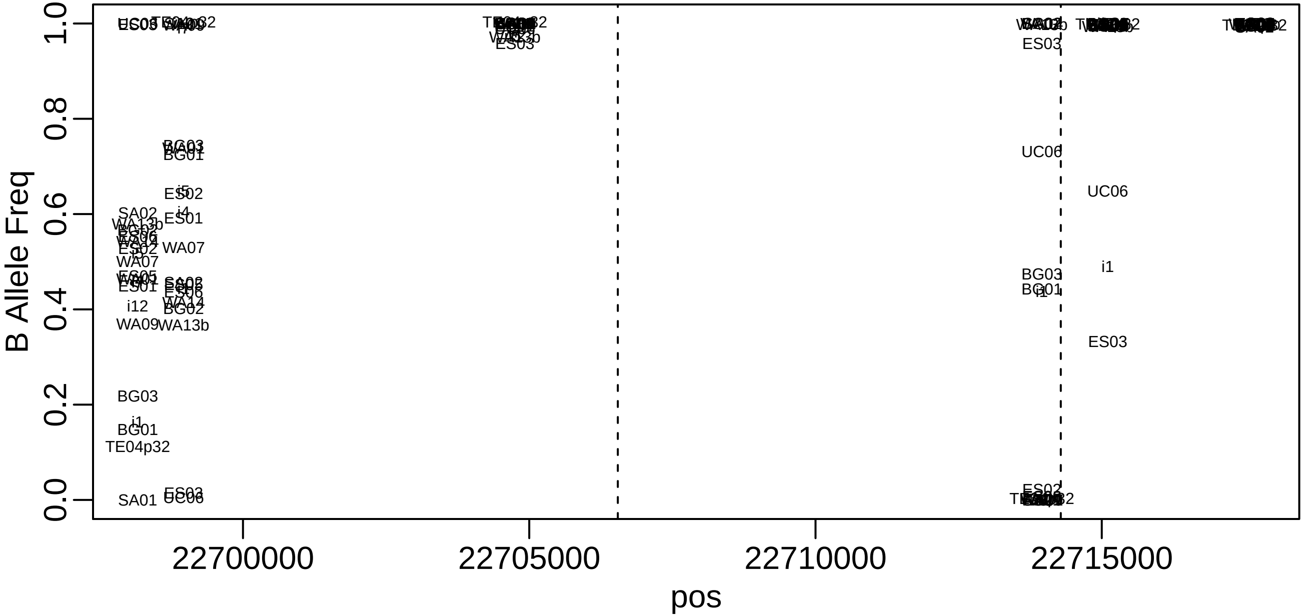

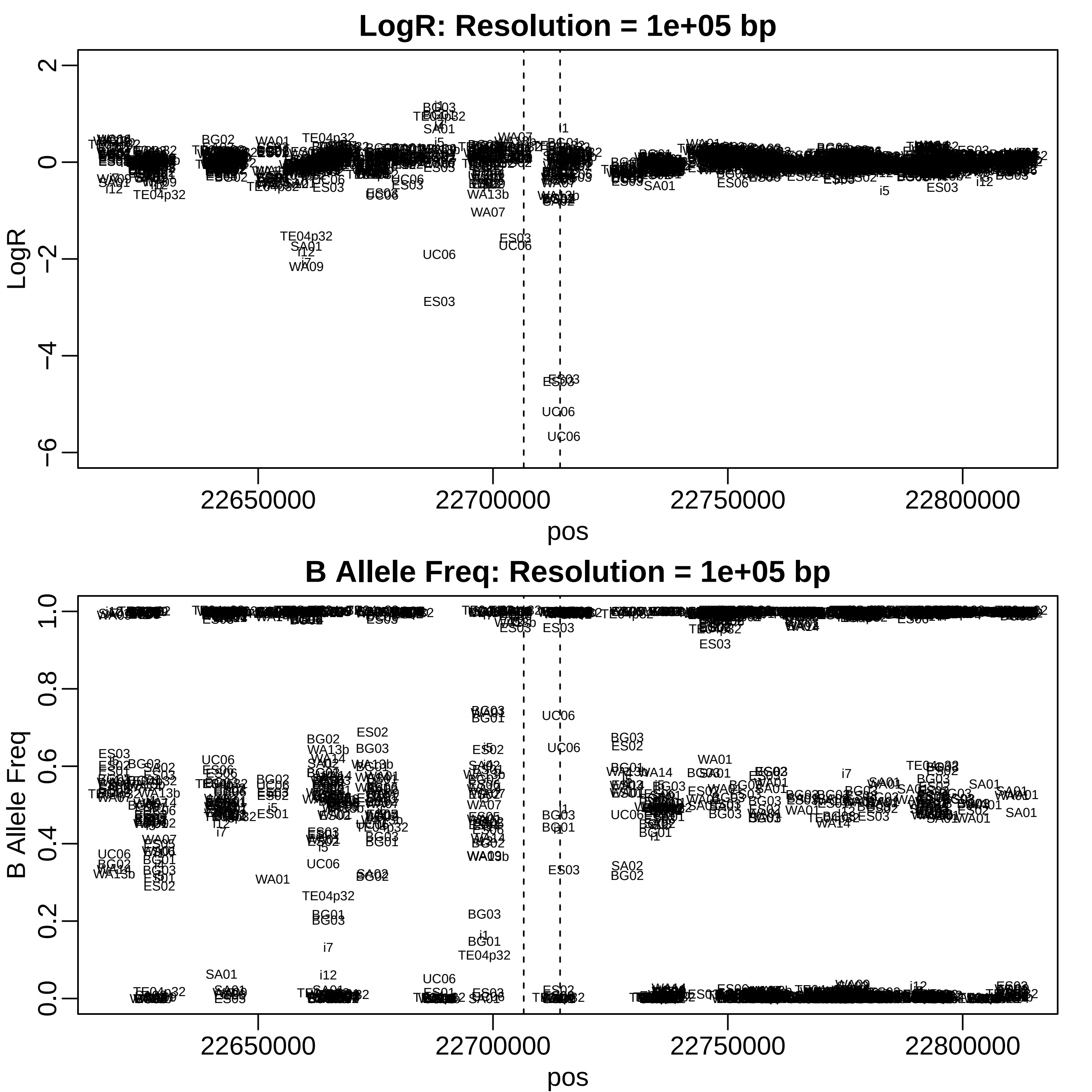

LogR: Resolution = 1e+05 bp
LogR
2
0
−2
−4
−6
22650000
22700000
22750000
22800000
pos
B Allele Freq: Resolution = 1e+05 bp
B Allele Freq
0.0
0.2
0.4
0.6
0.8
1.0
22650000
22700000
22750000
22800000
pos

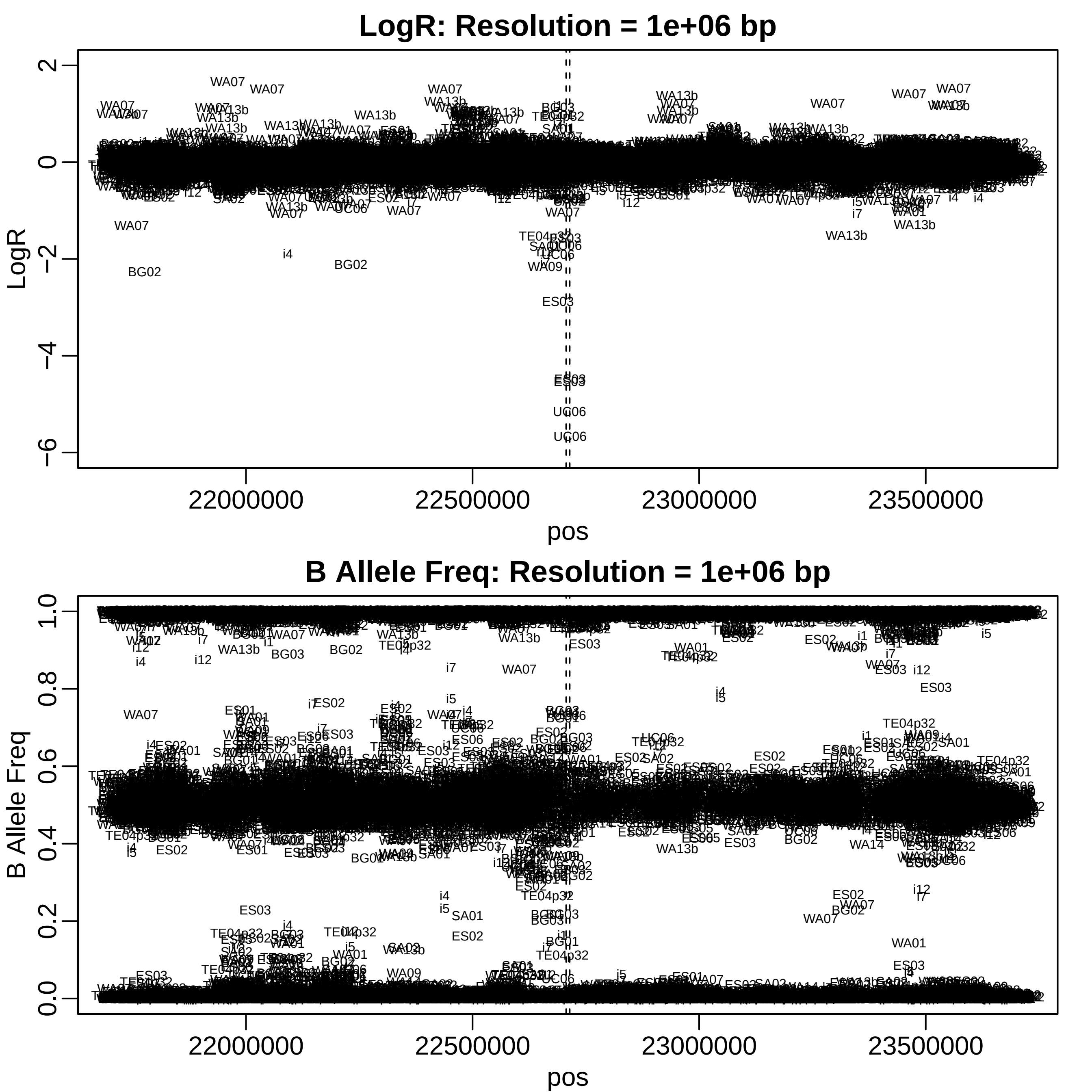

LogR: Resolution = 1e+06 bp
LogR
2
0
−2
−4
−6
22000000
22500000
23000000
23500000
pos
B Allele Freq: Resolution = 1e+06 bp
B Allele Freq
0.0
0.2
0.4
0.6
0.8
1.0
22000000
22500000
23000000
23500000
pos
